# Evaluation of a Chair-Mounted Passive Trunk Orthosis: A Pilot Study on Able-Bodied Subjects

**DOI:** 10.3390/s21248366

**Published:** 2021-12-15

**Authors:** Ahmad Zahid Rao, Muhammad Abul Hasan

**Affiliations:** 1 Department of Biomedical Engineering, NED University of Engineering and Technology, Karachi 75270, Pakistan; ahmadrao@neduet.edu.pk; 2 Neurocomputation Lab, National Center of Artificial Intelligence, Karachi 75270, Pakistan

**Keywords:** Duchenne muscular dystrophy (DMD), electromyography (EMG), exertion, orthosis, stability, trunk, usability

## Abstract

Trunk stability is important for adequate arm function due to their kinematic linkage. People with Duchenne muscular dystrophy (DMD) can benefit from trunk-assistive devices for seated daily activities, but existing devices limit trunk movement to forward bending. We developed a new trunk orthosis that has spring and pulley design. This study evaluated orthosis performance with 40 able-bodied subjects under with and without orthosis condition in 20 seated tasks for trunk rotation, forward bending, and side bending movements. Subjects adopted static posture in specific trunk orientation while their muscle activity was recorded. They also rated the subjective scales of perceived exertion and usability. A percent change in muscle activity for each task, due to orthosis use, is reported. Significant muscle activity reductions up to 31% and 65% were observed in lumbar and thoracic erector spinae muscles, respectively. Using three-way ANOVA, we found these reductions to be specific to the task direction and the choice of upper limb that is used to perform the asymmetric tasks. A total of 70% participants reported acceptable usability and ~1-point increase in exertion was found for maximum voluntary reaching with the orthosis. The outcomes of this study are promising, though tested on able-bodied subjects. Hence, orthosis mounted on wheelchairs should be further evaluated on DMD patients.

## 1. Introduction

The ability to regulate trunk movement and maintain an upright upper body is crucial when seated. This is because the human trunk is part of the kinematic chain that allow our hands to move in the functional workspace [1,2]. A seated person can only maximally use their upper limb when there is proper trunk flexibility. A partial or total loss of trunk control can happen due to neuromuscular conditions such as Duchenne muscular dystrophy (DMD) [3,4]. DMD is a progressive muscle-wasting disease that also affects the upper limb and trunk muscles, making it challenging to keep the body upright and stable. Consequently, it becomes difficult to catch oneself to prevent potential falls resulting from trunk movement in daily activities. An adequate trunk control is, therefore, critical for DMD patients in performing daily activities [5].

A DMD patient who is confined to a seated position in the wheelchair relies on trunk to perform upper limb activities in their reachable workspace [5,6]. As the weakness affects the upper limb muscles, the arm function needs to be supported by compensatory trunk movements to enhance the reachable workspace. In this attempt, the weak trunk muscles of DMD patients may cause a fall. Therefore, specifically designed trunk-assistive devices are required for seated persons that allow trunk bending and trunk rotations. Devices and studies for trunk postural control of people with DMD are scarce, while those that are developed have limitations in their capabilities.

A single degree of freedom (DoF) trunk-assistive device for people with DMD was developed and evaluated for trunk flexion and extension tasks [7]. The device comprises a gas spring and cam assembly that stores energy during trunk flexion and uses this energy to push the trunk in extension. The system is fixed to the user’s chair and has polycarbonate links connecting the front pad to the user’s chest. The device was found to reduce trunk muscle activation when maintaining a constant forward bent trunk posture. Another postural support device for DMD patients consists of electromechanical actuators [8]. The study evaluated the feasibility of various control modalities such as using the electromyography, the joystick, and the forces at chest or feet level. It is mounted on the chair and contacts the user’s trunk at the horizontal rod in front of the user. The rod then connects to each side of the chair where identical actuators are situated. The experiment consists of using each of the control modalities to perform a position-tracking task on the graphical user interface. Time- and distance-based performance metrics found all modalities as practical, while joystick was the most favorable. However, this device limits the movement to a single DoF.

The existing devices allow movement in one DoF only; however, most daily activities involve general movement, traversing multiple anatomical planes. Incorporating more DoF in the trunk-assistive device can enable the user to cover most of their functional workspace from a seated position. We developed a new orthosis, the Chair-Mounted Passive Trunk Orthosis (CMPTO), which has four DoF and is based on a spring-and-pulley design. This orthosis will provide antigravity assistance for trunk stability which may allow DMD patients to move their trunk as required for their daily activities without worrying about potential falls. Furthermore, we tested and analyzed the effect of CMPTO on trunk muscle activity during various seated postures of unsupported upright trunk and maximum trunk sway, along multiple directions in the horizontal plane. When using CMPTO, we expect an increased muscle activity in the unsupported upright trunk postures and a decrease in muscle activity for maximum trunk sway postures.

## 2. Materials and Methods

### 2.1. Participants

The sample size was computed through the G * Power software (version 3.1.9.7, available online from the University of Dusseldorf, Germany) [9]. The required sample size was thirty-four, based upon a priori analysis and the parameter values of 0.05 for the significance level, 0.80 for the statistical power, and 0.50 for a medium effect size with two-tailed one-sample case. However, for more prominent differences, a convenience sample of forty (40) able-bodied male adults was recruited at this phase of the study (mean ± std, age: 261.75 ± 37.51 months, mass: 69.39 ± 15.92 kg, height: 173.60 ± 6.49 cm, body mass index: 22.92 ± 4.68 kg/m^2^) from the university and local community. Exclusion criteria involved any current or recent (past 12 months) pain, weakness, disease, or condition that may hinder the control of the trunk, or the movement itself. This research complied with the tenets of the Declaration of Helsinki and was approved by the Research Ethics Committee of NED University of Engineering and Technology (approval no. ASRB/878). Informed consent was obtained from each participant.

### 2.2. Chair-Mounted Passive Trunk Orthosis (CMPTO)

**Figure 1:**
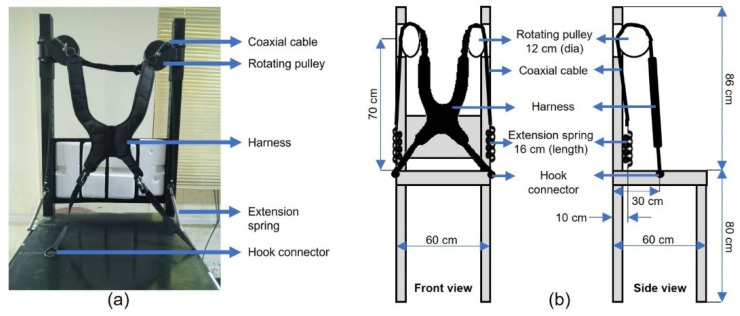
The chair-mounted passive trunk orthosis (CMPTO). (**a**) CMPTO photograph as it is mounted on experimental chair; (**b**) CMPTO schematics for front view (left) and side view (right).

The CMPTO is developed with a padded chest harness, two rotating pulleys, and two extension springs connected in series via inelastic coaxial cables (Figure 1). The smallest spring stiffness used for load-lifting tasks in standing position for a trunk-assistive device was 0.3 N/mm [10]. Keeping in mind that the seated tasks require less trunk force, we initially chose three springs having high (0.3906 N/mm), medium (0.1435 N/mm), and low (0.0514 N/mm) stiffnesses. Anecdotal feedback from five volunteers found high stiffness springs were too restricting for the flexion/extension movement, while the low stiffness springs were too easy to move, making them ineffective for trunk stability. The spring selected had a stiffness of 0.1435 N/mm, the material was stainless steel 304, length 15.4 cm, diameter 15 mm, and wire thickness 1.4 mm. The padded chest harness contacts the seated user and has four straps extending from its edges. The lower two straps are fixed to either side of the chair via hooks (30 cm from rear end), while the upper two straps are attached to inelastic coaxial cables. These cables go over rotating pulleys (diameter: 12 cm), which are mounted on the vertical rods of the extended backrest of the chair above the user’s either shoulder. The rotating pulleys allow CMTPO to be moved freely along the horizontal plane with four DoF (two from each rotating pulley). The end of each cable links to an extension spring fixed on the side of the chair (10 cm from rear end). The seating platform of the chair has dimensions of 60 cm × 60 cm and is 80 cm high from the floor. The seat height was kept high only for the experimental purpose to mimic the condition of wheelchair-bound DMD patients as they are not able to use their feet to support the reaching movements [11]. This restricted the able-bodied subjects to use foot support for trunk stability during the experimental tasks. The extended backrest goes 86 cm above the seat, while both rotating pulleys are fixed at 70 cm above the seat.

### 2.3. Procedure

**Figure 2:**
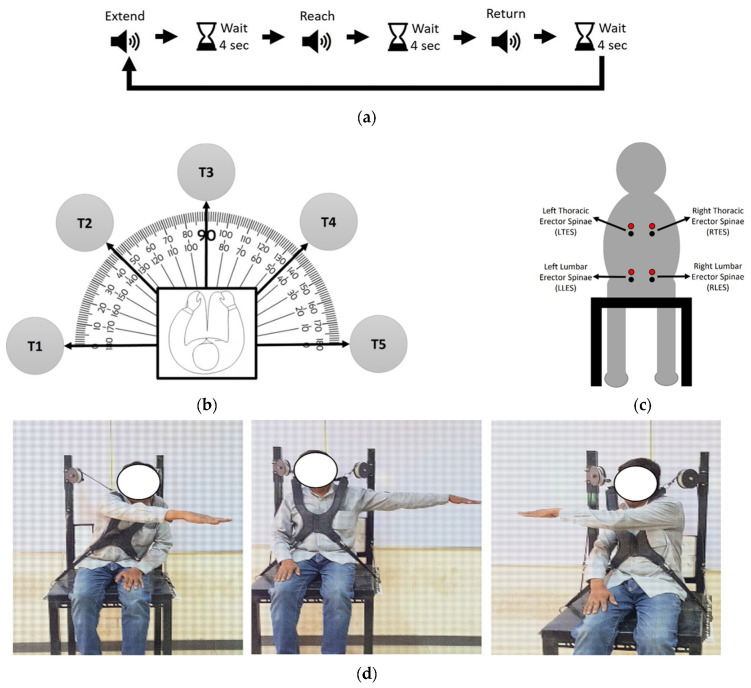
Experimental procedure. (**a**) The sequence of events in each task. The task started with the audio instruction of extending arm towards target (Extend), followed by a pause of 4 s, then audio instruction of reaching for the target (Reach), followed by a pause of 4 s, then audio instruction of returning to neutral position (Return), followed by a pause of 4 s. Audio instructions for the next task are then generated in the same manner. (**b**) The location of targets with respect to the seated participant (top view). Five targets, named T1, T2, T3, T4, and T5, are placed relative to the chair at precisely measured (from left side) angles of 0°, 45°, 90°, 135°, and 180°, respectively. All targets are placed at a sufficient distance from the chair to avoid obstruction during the task. (**c**) The bipolar, positive (red), and negative (black) electrode placement for each muscle of interest on the seated participant (rear view). (**d**) Examples of static posture maintained by participant with orthosis when oriented towards targets (mentioned in parenthesis). Left: Reach with right limb at 135° (T2); Center: Extend with left limb at 0° (T1); Right: Reach with left limb at 180° (T5).

Participants’ demographic data (age, height, and weight) was obtained followed by skin preparation and attachment of surface EMG electrodes over marked locations for muscles of interest. The participants sat on the chair and performed three (03) trials of tasks under each of the two conditions, i.e., with orthosis and without orthosis. The order of both conditions was systematically varied across subjects so that half of the subjects performed tasks with orthosis first and the rest performed tasks without orthosis first. A single trial consisted of 10 tasks in random order guided by timed audio instructions. Each task comprised three stages: extend the upper limb with an unsupported upright trunk (Extend), perform maximal trunk sway from the neutral upright posture (Reach), and return to resting upright position (Return), as depicted in Figure 2a. A four (04)-second pause was given after each audio to let the participant follow instruction. Appropriate rest was given between the trials to prevent any potential fatigue effects. The tasks were performed with both upper limbs in five (05) fixed orientations at 0°, 45°, 90°, 135°, and 180°, measured with respect to the fully extended horizontally abducted upper limb such that 0° with right limb is same direction as 180° with left limb, and so on. From the participant’s perspective, however, these orientations were called T1 to T5, as shown in Figure 2b.

The participants were verbally instructed to extend upper limb at shoulder height, maintain upright trunk during Extend stage, and make movements along the horizontal plane towards the target in Reach stage, while adopting their natural approach as in daily life routine. For participants’ ease of visualization, wooden targets (height: 124 cm from the ground) bearing tags of target name were placed at precisely calculated orientations. To avoid interference with the movement, these targets were at 1.5 times the arm length distance away from the chair (range: 105 cm to 122 cm). Figure 2d shows a few example postures in the experiment. After all the trials were completed under both conditions, the participants were asked to fill out the usability (SUS) [12] and the perceived exertion (Borg CR-10) [13] questionnaires.

### 2.4. Measurements

#### 2.4.1. Muscle Activity

The muscle activity during the experiment was recorded at 2048 Hz sampling frequency using an EMG device (model: Mobi, company: Twente Medical Systems International (TMSi), Oldenzaal, The Netherlands, manufacture year: 2016, certification: CE marked, intended use: research or clinical applications). During each task, the EMG was measured for 4 s for Extend and Reach stages. Muscle activity was measured over four posterior trunk muscle locations: bilateral thoracic erector spinae (RTES and LTES) muscles and bilateral lumbar erector spinae (RLES and LLES) muscles (Figure 2c).

The electrodes for TES muscles were located 3 cm bilaterally to the T9 spinous process while for the LES muscles, at 3 cm bilaterally to the L4 spinous process, and ground electrode over right iliac crest, following the SENIAM guidelines [14]. Skin was lightly abraded and cleaned using 70% isopropyl alcohol swabs for improved electrical connection. Pregelled bipolar Ag/AgCl electrodes having adhesive padding of 5 cm in diameter were used for secure contact during movement.

#### 2.4.2. Perceived Exertion

The user’s ratings of perceived exertion (RPE) assessed CMPTO’s effect on the intensity of physical activity during maximal reach task in all tested directions. Each participant rated 20 RPE scores (for five directions with two limbs under two orthosis conditions). The participants were instructed to remain vigilant in their perception of exertion in Reach stages during the experiment. They rated their perceived exertion using the Borg Category Ratio-10 (CR-10) [13] questionnaire that has a simple numerical list with exertion descriptors ranging from 0 (rest) to 10 (maximal).

#### 2.4.3. CMPTO Usability

The usability ratings measured CMPTO’s potential ease of use. The participants filled out the System Usability Scale (SUS) [12] questionnaire for CMPTO at the end of the experimental trials. SUS comprised of 10 standard questions regarding CMPTO for which the participants must select among five Likert-scale response options ranging from strongly disagree (1) to strongly agree (5), based on their level of agreement.

### 2.5. Data Processing

All data processing was carried out using MATLAB software (model: R2018b, company: MathWorks, Natick, MA, USA, manufacture year: 2018). A band-pass filter of 20–450 Hz was applied to obtain the desired EMG signal. Power line noise and electrocardiography artifacts were removed using a notch filter of 50 Hz and a high-pass filter of 30 Hz [15,16], respectively. All filters were third-order bidirectional IIR Butterworth filters. For each trial, the EMG signal was segmented into 4 s epochs corresponding to the task stages of Extend and Reach. To eliminate movement artifacts and represent muscle activity during isometric contraction for static posture, a 2 s data portion from the epochs was further analyzed. This was from 2 to 4 s from the onset of Extend stage, and from 1.5 to 3.5 s from the onset of Reach stage.

The resulting signals were rectified, and their mean absolute value (MAV) envelopes were computed to determine the signal amplitude, using a 100-millisecond sliding window with 100 samples overlap. This resulted in 40 MAVs for each second of the epoch for moving average purpose. MAV is defined as the absolute value of each sample (*x*) in the dataset, where *n* is the total number of samples:

(1) $$\mathrm{MAV}=\frac{1}{n}\sum_{i\;=\;1}^{n}\left|x_{i}\right|$$

Participants performed three trials of each task in random order under both conditions: orthosis and without orthosis. Muscle activity in each trial of orthosis condition was percentage-normalized with respect to average muscle activity from all three trials of without orthosis condition for each subject:

(2) $$nEMG_{subject}=\frac{1}{3}\overset{3}{\underset{\mathrm{trial}\;=\;1}{\sum }}\left(\frac{orthosisMAV_{trial}-averageMAV}{averageMAV}\right)\times 100\%$$

where *averageMAV* is given by:

(3) $$averageMAV=\frac{1}{3}\overset{3}{\underset{\mathrm{trial}\;=\;1}{\sum }}\left(without\_orthosisMAV_{trial}\right)$$

Hence, a normalized muscle activity (*nEMG*) of zero percent meant no difference in the muscle activity between both conditions, while a positive normalized value indicated an increase in muscle activity when the CMPTO was used. The outcome variable was the average value (*n* = 40) of normalized muscle activity in the isometric Extend and Reach stages.

To evaluate subjective measures, the ratings on questionnaires were considered. The scores of each SUS question were added to obtain a rating for usability of the CMPTO, where a 70+ score is considered acceptable. The number of participants scoring CMPTO as acceptable was reported. To find the mean effect of CMPTO on RPE during every task, each of the 20 Borg CR-10 ratings were separately averaged across all participants.

### 2.6. Statistical Analysis

Separate, three-way analysis of variance (ANOVA) for each stage was performed to find the effect of independent variables on dependent variable. The dependent variable was the normalized muscle activity, while independent variables were Muscle, Task Orientation, and Limb Used in task. In addition to investigating the main factor effects, the interaction effects between factors were obtained. *p*-values were corrected using the Holm–Bonferroni procedure to control the familywise error rate for multiple comparisons. Effect sizes (eta-squared η^2^) were calculated, from ANOVA results, as the ratio between effect’s sum of squares and the total sum of squares. The one-sample *t*-tests were applied on the percentage-normalized EMG data to determine statistically significant changes in muscle activity between orthosis and without orthosis conditions. This was performed for both the stages in all the 10 tasks for each muscle. Post hoc paired comparisons were carried out using false discovery rate procedure to control type I error with Benjamini–Hochberg (B–H) procedure. The nonparametric Wilcoxon signed rank test was applied on the ratings of perceived exertion in each task under both conditions to determine the statistically significant differences. All statistical analysis were performed on MATLAB software, and significance level of *p* < 0.05 was kept for all statistical tests.

## 3. Results

### 3.1. Factors Contributing toward Varied Muscle Activity in Extend and Reach Stages

Table 1 shows the summarized ANOVA results for both stages. The F-statistic, *p*-value corrected for multiple comparisons, and eta^2^ for effect size are given together with degrees of freedom for each effect. These represent how the muscle activity varies with the factors in each effect. The main effects of Muscle, Limb Used, and Task Orientation factors were not significant for the Extend and Reach stages. However, the interaction effects of “Muscle x Limb Used” (Extend: F = 30.03, *p* < 0.001, η^2^ = 0.051; Reach: F = 14.12, *p* < 0.001, η^2^ = 0.023) and “Muscle x Task Orientation” (Extend: F = 2.34, *p* = 0.034, η^2^ = 0.016; Reach: F = 10.79, *p* < 0.001, η^2^ = 0.068) are significant under both conditions. The effect sizes show a greater effect of Muscle × Limb Used interaction for the Extend stage, while greater effect of Muscle × Task Orientation interaction for the Reach stage.

### 3.2. Effect of CMPTO on Muscle Activity

**Figure 3:**
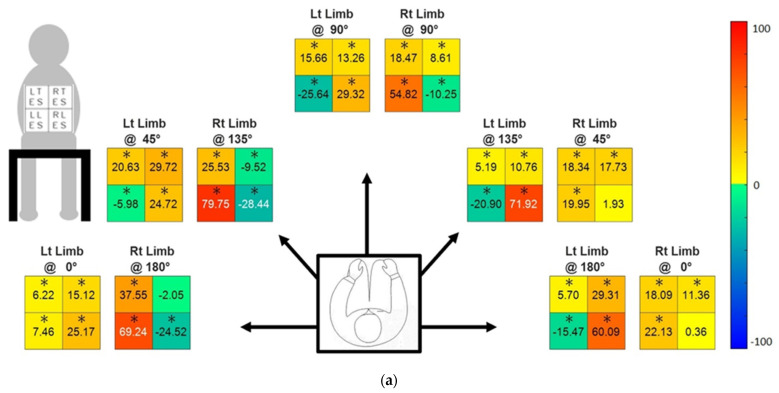

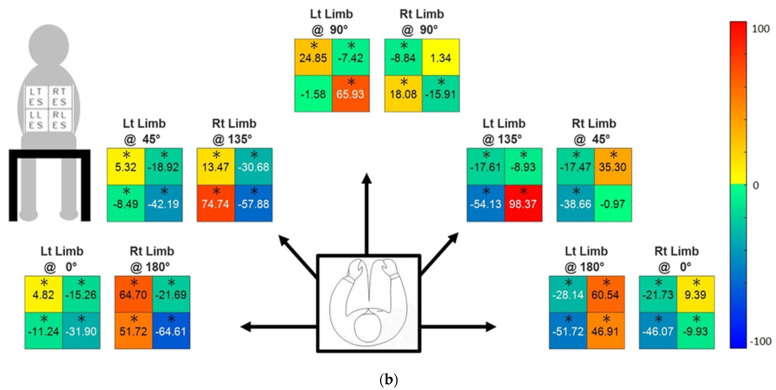
Percent change for (**a**) Extend stage and (**b**) Reach stage, in the EMG mean absolute value (MAV) due to CMPTO. The four values within each heatmap represent each of the four muscles (LTES, RTES, LLES, and RLES muscles), whose order is shown near the top left corner. The five pairs of heatmaps represent the five orientations (0°, 45°, 90°, 135°, and 180°). Each pair of heatmaps represent the data when using either limb (Rt: right, Lt: left). The scale bar on the right side gives the range of intensities with corresponding numeric values, as used in heatmaps. An asterisk over individual value indicates a significant change in muscle activity due to CMPTO use (*p* = 0.05).

The percentage changes in muscle activities due to CMPTO use at Extend and Reach stages are reported using heatmaps in Figure 3a,b, respectively.

Each of the 10 heatmaps represent the muscles’ average (*n* = 40 participants) percentage change in activity due to orthosis with each limb along each task orientation, expressed between −100 to +100 percent change. Within each heatmap, there are four boxes that represent the four muscles analyzed. The top two boxes represent TES muscles while the bottom two represent LES muscles; the right two boxes represent muscles towards the right side when viewed from the rear, while the left two represent those towards the left side of the body. A positive heatmap value (towards red intensity) indicates a percentage increase in muscle activity, while a negative value (towards blue intensity) indicates a percentage decrease in muscle activity when using the CMPTO. A significant percentage difference of muscle activity with CMPTO is denoted by an asterisk over the values in heatmap boxes.

Upright trunk posture (Extend stage): The CMPTO causes a significant reduction in the ipsilateral LES muscle activity for task orientations with horizontally adducted limb in Extend stage (Figure 3a). These reductions are 10.25%, 28.44%, and 24.52% with right limb, and 25.64%, 20.90%, and 15.47% with left limb, at 90°, 135°, and 180° task orientations, respectively. Apart from the reductions, a trend of increased muscle activity is present generally.

Maximum voluntary excursions (Reach stage): Further patterns of reductions in muscle activity due to CMPTO are observed in Reach stage (Figure 3b). At 0° and 45° orientations, the reduction in muscle activity of up to 46%, 22%, and 11% is observed in contralateral LES muscle, contralateral TES muscle, and ipsilateral LES muscle, respectively. At 90° orientation, a reduction of up to 16% and 9% is observed in ipsilateral LES muscle and contralateral TES muscle, respectively. At 135° and 180° orientations, a reduction of up to 65% and 31% is observed in contralateral LES muscle and contralateral TES muscle, respectively. The increase in muscle activity due to CMPTO is observed in ipsilateral TES muscle at 0° and 45° orientations, in ipsilateral TES and contralateral LES muscles at 90° orientation, while in contralateral TES and contralateral LES muscles at 135° and 180° task orientations.

### 3.3. Perceived Exertion

**Figure 4:**
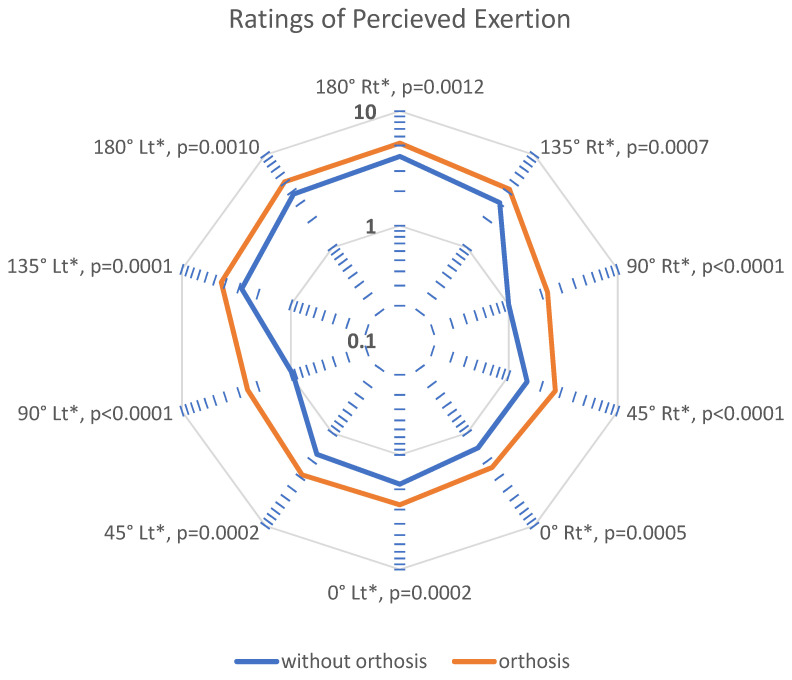
Ratings of perceived exertion (RPE) using radar plot in Reach stage, i.e., maximum trunk sway from neutral position. The blue line represents without orthosis condition, while the orange line represents orthosis condition. Each axis of the plot represents the 10 tasks, i.e., at five orientations (0°, 45°, 90°, 135°, and 180°) using two limbs (Rt: right limb, Lt: left limb). The axes are plotted using logarithmic scale between 0 and 10. An asterisk in axis label indicates a significant difference in the RPE rating between orthosis and without orthosis conditions, and the *p*-values are also shown.

Figure 4 shows the mean RPE ratings (n = 40 participants) of Borg CR-10 scale during Reach stage under each condition, at each orientation, and with either upper limb. The 10 axis of radar plot represent the 10 tasks in the experiment. The axes have logarithmic scale to reduce skewness towards large values. The RPE for orthosis condition are higher in all the tasks. The RPEs for orientations with horizontally abducted upper limb (0°, 45°) are lower as compared to those with horizontally adducted upper limb (135°, 180°). The difference between the two data points, from each condition, on the axis is largest for 90° orientation, followed by horizontally abducted orientation (0°, 45°), and horizontally adducted orientation (135°, 180°). These differences between the two data points are significant for all 10 tasks.

### 3.4. CMPTO Usability

A total of 28 of the 40 participants (70%) rated SUS score above the acceptable usability criterion of 70, while 11 participants (27.5%) rated between 60–70 score. Table 2 shows the score for each SUS question where the absolute difference indicates the degree of deviation of the participants’ average score from an ideal score (leading to SUS score of 100) against each SUS question. Responses to all questions have average score within 1-point difference, except for one where it is 3-point difference.

## 4. Discussion

The goal of this study was to evaluate the effect of CMPTO which allows performing seated tasks in functional workspace along the horizontal plane. These seated tasks comprised the Extend and Reach stages, which correspond to the unsupported upright trunk and the maximal trunk sway, respectively. The tasks performed in Extend and Reach stages resemble trunk stability and trunk fall situation for DMD patients, respectively. The increased muscle activity in Extend stage and decreased muscle activity in Reach stage, along with acceptable usability score and slight increase in RPE with CMPTO in able-bodied subjects, imply feasibility for further testing in DMD patients.

### 4.1. Muscle Activity with CMPTO

The literature for the evaluation of trunk orthosis contains several experimental tasks that involve maintaining a trunk flexion posture, or functional tasks such as lifting and lowering of weight, walking, and sit-to-stand [7,8,17,18]. These studies include devices for standing applications as well as those for sitting applications, such as for DMD patients. Most of these tasks involve movement in a single plane, while a few studies require trunk rotations at some orientations [19,20,21]. However, our experiment involved several tasks differing in trunk orientations for a closer representation of the entire seated trunk range of motion. The trunk muscles activity varied with the trunk orientations and with the choice of upper limb used to perform the task, because the nature of the task placed variable demands on trunk subsegments [22].

In Extend stage, the participants had to maintain an upright trunk posture with one arm extended in a particular direction, without taking support from any other means such as the backrest. Therefore, a minimal trunk muscle activity is expected in without orthosis condition for this stage [23,24]. The use of CMPTO, generally, increases the trunk muscle activity which can be attributed to the pulling force from the springs in the orthosis. As the backrest is away from the trunk, an increased muscle activity generates the additional force required to overcome that spring’s pulling force to remain stationary. Although the participants overcame this force in both stages, its effect is prominent in the Extend stage because the task otherwise required a slight effort.

In Reach stage, the participant had to maximally move the trunk away from the upright position without falling. It is believed that coactivation, which is the concurrent contraction of agonist and antagonist muscles around a joint for providing joint stability, takes place between the ipsilateral and contralateral ES muscles. It is well known that the trunk lateral bending is facilitated by ipsilateral ES muscle activity and gravity, while hindered by contralateral ES muscle to control the rate and amount of bending [25]. The CMPTO assists the role of contralateral ES muscle, leading to a general decrease in muscle activity as compared to without orthosis condition. Interestingly, the reduced trunk muscle activity is in line with previous studies on trunk-assistive devices where the reductions ranged from 22% [18] to 38% [17] in ES muscles for static forward bending tasks, while from 28% [20] to 34% [21] for tasks involving trunk rotations. However, the muscle activity reductions with CMPTO for the Reach stage are higher than those reported in these studies. Moreover, the utilization of trunk inertia against orthosis to maintain static posture may also contribute to a reduced demand of muscle force. This can be beneficial to increase user’s endurance and decrease muscle fatigue and mechanical loading on the spinal column, thus reducing the risk of developing lower back disorders and encouraging the movements involving trunk rotations. Finally, it can be argued that the muscle activity reduction may arise from a decreased trunk flexion angle in Reach stage, where the trunk did not move to the same extent with CMPTO as without it. However, it should be kept in mind that the task required the participants to apply their maximum effort in both conditions. Consequently, it can be inferred that the similar level of muscle activity was generated in both conditions.

### 4.2. User Experience with CMPTO

The user experience was reflected by the usability and perceived exertion responses. The RPE in Reach stage represents the participants’ perception when they are simultaneously working against the CMPTO springs and maintaining balance in static posture. The ratings, therefore, are expected to be higher with the CMPTO. Such response has also been observed previously for different trunk orthosis [19,21]. It should be noteworthy that these ratings reflect the maximum possible exertion levels required for the experimental design, whereas most daily life activities would require lower exertions.

The CMPTO springs apply considerable pulling force at 90° orientation (forward trunk movement). Therefore, the difference in exertion levels between both conditions is the greatest at 90° orientation, compared to other orientations for the same upper limb. Furthermore, the tasks requiring horizontally adducted arm (at 135° and 180° orientations) are perceived as difficult as this involves a higher degree of trunk rotation, demanding increased effort [26]. In these cases, the spring towards the upper limb involved in the task applies higher resistive force. Thus, higher exertion levels are found in with and without orthosis condition, causing a small difference in perceived exertions between these conditions. In contrast, the tasks requiring horizontally abducted arm (at 0° and 45° orientations) have the resistive force applied by the spring towards the opposite upper limb as the one involved in task. Therefore, these tasks are perceived lighter since the upper limb used in the task is unobstructed. Nevertheless, it should be kept in mind that the subjective nature of RPE ratings may be influenced by intrinsic factors such as participants’ prior experience with spring-based devices or their involvement in exercise or other exertion activities [27].

Importantly, most of the participants rated the CMPTO as having acceptable usability. Nevertheless, the few participants who rated the orthosis below the criterion saw technical assistance necessary to help with all the information. Their usability ratings may have been negatively influenced by the time taken for the experimental setup, the assistance needed to don and doff the device, and the experimental procedure instructions [28]. However, during routine tasks in a home setting, these factors would be eliminated as specific task instructions or testing equipment, such as sensor placement, would not be required. Moreover, comfort with CMPTO may be enhanced by an improved pressure distribution scheme and a personalized height adjustment of the device. The design of harness, primarily covering the abdomen area, was influenced by the need to access posterior trunk skin surface for EMG signal acquisition. It is likely that an improved harness design covering more trunk area may better distribute resistive forces and hence decrease pressure.

### 4.3. Implications for DMD Patients

The orthosis is designed specifically to be used by DMD patients. This pilot study recruited able-bodied subjects for the initial evaluation of the CMPTO. The experimental tasks carried out for the evaluation of trunk-assistive devices normally comprise daily activities [29] or frequently encountered postures [7]. In contrast, our experiment involved tasks that required unsupported upright sitting and maximum trunk sway. The purpose of such tasks was to mimic the circumstances of DMD patients when they are sitting on the wheelchair and are making strenuous effort with their upper limb and trunk to reach out for objects in daily routine. The outcomes from able-bodied subjects can have implications for DMD patients so that they may be encouraged to use CMPTO.

The muscle activity increased with CMPTO in the Extend stage due to the spring’s pulling force which needed to be overcome to maintain an upright posture. The presence of this pulling force has importance for DMD patients, as this will stabilize their trunk even during quiet sitting. In other words, this pulling force will attenuate the small trunk perturbations that arise from upper limb movement. The muscle activity reductions observed when using the CMPTO in the Reach stage suggests (a) the involvement of inertia in maintaining posture and (b) a reduced trunk flexion angle, as discussed in Section 4.1. From the patient’s perspective, these may propose that while inertia can be utilized in performing trunk movement with CMPTO, the orthosis will effectively work as an antigravity support and its progressive resistance will keep the trunk close to the chair backrest. Thus, the CMPTO can keep the trunk stable and prevent potential falls associated with larger displacement of trunk center of mass [30,31,32].

The reported RPEs in Reach stage show that despite the maximal effort by the participants, there are small differences in exertions when using the CMPTO. From the patients’ outlook, this could be an encouraging finding because it implies that, in case the patient loses their trunk balance, the orthosis will not let them fall from the chair and will not cause much discomfort. Hence, they can perform their daily activities without worrying about the possibility of falling due to weak trunk muscles. The favorable usability results reflect upon the aspects of CMPTO sturdiness and its ease of use and imply that the DMD patients can use the CMPTO to enhance their upper limb function without being concerned about its durability [33].

### 4.4. Other Applications of CMPTO

A partial or total loss of trunk control is also common in other neuromuscular conditions such as multiple sclerosis [34] and cerebral palsy [35]. In these cases, the body orientation and movement are disturbed, leading to frequent falls and injuries. Thus, the CMPTO may have potential application for people with these trunk disorders. Moreover, the design concept of CMPTO, with slight modifications, may be used as a trunk-assistive device for people with Parkinson’s disease [36], stroke [37], brain injury [38], and tetraplegia [39].

### 4.5. Study Limitations

One of the limitations was having few muscles for EMG analysis, though the muscles most widely studied for evaluations of trunk-assistive devices were included. Still, more trunk muscles can be studied to observe how CMPTO can affect their activation levels. Furthermore, whereas this study focused on the muscle activity and user experience with CMPTO, yet another interesting domain for future work is the kinematic analysis for investigating the movement patterns and their variations due to the use of CMPTO. It can be argued that only male participants were recruited in this study, but this is the case because the genetic nature of DMD affects only the male population. However, it is recommended that large studies should also include females as they may be carriers and, in rare cases, may experience muscle weakness [40]. Another limitation can be the short period of study. Since the CMPTO is expected to be used by patients over extended periods of time, a longitudinal study design may reflect upon the exertion and fatigue effects due to its prolonged use. Lastly, the frictional effects of CMPTO can be measured as well, to know exactly how much friction is present for each directional task and subsequently be reduced to improve the user experience.

## 5. Conclusions

We developed a spring-and-pulley-based orthosis, called CMPTO. We tested the orthosis in various seated tasks with able-bodied subjects for muscle activity in Extend and Reach stages, the user’s perceived exertion, and device usability. The increased muscle activity in Extend stage signifies the presence of antigravity assistive pulling force by the CMPTO. The decreased muscle activity and the increased perceived exertion in the Reach stage shows CMPTO usefulness in keeping the trunk close to the backrest as the user applies force against the orthosis. These outcomes, along with good usability score, show implication for testing CMPTO with DMD patients.

## Figures and Tables

**Table 1:** Summary of ANOVA results regarding main and interaction effects of Muscle, Limb Used, and Task Orientation factors on Extend and Reach stages’ muscle activity. F statistic, *p*-value, and effect size are given, and statistically significant results are bold-typed.

Source	df	Extend			Reach		
		F	*p*	η^2^	F	*p*	η^2^
Muscle	3	0.36	1.000	0.001	0.51	0.954	0.001
Limb Used	1	0.09	1.000	<0.001	0.51	0.954	<0.001
Task Orientation	4	0.11	1.000	<0.001	1.31	0.795	0.003
Muscle × Limb Used	3	**30.03**	**<0.001**	**0.051**	**14.12**	**<0.001**	**0.023**
Muscle × Task Orientation	12	**2.34**	**0.034**	**0.016**	**10.79**	**<0.001**	**0.068**
Limb Used × Task Orientation	4	0.46	1.000	0.001	3.19	0.064	0.007
Muscle × Limb Used × Task Orientation	12	0.73	1.000	0.005	1.72	0.228	0.012

**Table 2:** SUS questions and average scores (*n* = 40). Each question was scored on Likert scale from 1 to 5. Questions regarding positive aspects of CMPTO had ideal score of 5, while those regarding negative aspects of CMPTO had ideal score of 1. Absolute difference is found between average score and ideal score.

#	System Usability Scale Questions	Average Score	Ideal Score	Absolute Difference
1	I think that I would like to use the orthosis frequently	1.975	5	3.025
2	I found the orthosis unnecessarily complex	1.875	1	0.875
3	I thought the orthosis was easy to use	4.350	5	0.650
4	I think that I would need the support of a technical person to be able to use the orthosis	2.000	1	1.000
5	I found the various functions in the orthosis were well integrated	4.175	5	0.825
6	I thought there was too much inconsistency in the orthosis	1.500	1	0.500
7	I would imagine that most people would learn to use the orthosis very quickly	4.650	5	0.350
8	I found the orthosis very cumbersome (awkward) to use	1.950	1	0.950
9	I felt very confident using the orthosis	4.325	5	0.675
10	I needed to learn a lot of things before I could get going with the orthosis	1.925	1	0.925

## Data Availability

Not applicable.
